# Mechanisms of Cardiovascular Disorders in Patients With Chronic Kidney Disease: A Process Related to Accelerated Senescence

**DOI:** 10.3389/fcell.2020.00185

**Published:** 2020-03-20

**Authors:** Julia Carracedo, Matilde Alique, Carmen Vida, Guillermo Bodega, Noemí Ceprián, Enrique Morales, Manuel Praga, Patricia de Sequera, Rafael Ramírez

**Affiliations:** ^1^Departamento de Genética, Fisiología y Microbiología, Universidad Complutense/Instituto de Investigación Sanitaria Hospital 12 de Octubre (imas12), Madrid, Spain; ^2^Departamento Biología de Sistemas, Facultad de Medicina y Ciencias de la Salud (IRYCIS), Universidad de Alcalá, Alcalá de Henares, Madrid, Spain; ^3^Departamento de Biomedicina y Biotecnología, Facultad de Biología, Química y Ciencias Ambientales, Universidad de Alcalá, Alcalá de Henares, Spain; ^4^Departamento de Nefrología, Hospital Universitario 12 de Octubre/Instituto de Investigación Sanitaria Hospital 12 de Octubre (imas12), Madrid, Spain; ^5^Departamento de Medicina, Universidad Complutense de Madrid, Madrid, Spain; ^6^Sección de Nefrología, Hospital Universitario Infanta Leonor, Madrid, Spain

**Keywords:** chronic kidney disease, cardiovascular diseases, reactive oxygen species, extracellular vesicles, microRNAs, cellular senescence, epigenetic alterations, atherosclerosis

## Abstract

Cardiovascular diseases (CVDs), especially those involving a systemic inflammatory process such as atherosclerosis, remain the leading cause of morbidity and mortality in patients with chronic kidney disease (CKD). CKD is a systemic condition affecting approximately 10% of the general population. The prevalence of CKD has increased over the past decades because of the aging of the population worldwide. Indeed, CVDs in patients with CKD constitute a premature form of CVD observed in the general population. Multiple studies indicate that patients with renal disease undergo accelerated aging, which precipitates the appearance of pathologies, including CVDs, usually associated with advanced age. In this review, we discuss several aspects that characterize CKD-associated CVDs, such as etiopathogenic elements that CKD patients share with the general population, changes in the cellular balance of reactive oxygen species (ROS), and the associated process of cellular senescence. Uremia-associated aging is linked with numerous changes at the cellular and molecular level. These changes are similar to those observed in the normal process of physiologic aging. We also discuss new perspectives in the study of CKD-associated CVDs and epigenetic alterations in intercellular signaling, mediated by microRNAs and/or extracellular vesicles (EVs), which promote vascular damage and subsequent development of CVD. Understanding the processes and factors involved in accelerated senescence and other abnormal intercellular signaling will identify new therapeutic targets and lead to improved methods of diagnosis and monitoring for patients with CKD-associated CVDs.

## Introduction

Chronic kidney disease (CKD) is defined as a systemic pathology that affects approximately 10% of the population; however, data showed differences in the prevalence of CKD between countries (Hill et al., 2016). The prevalence of CKD has increased markedly over the past decades due to aging of the population worldwide and increase in incidence of diabetes mellitus, which has become the primary cause of CKD. Nowadays, CKD is considered a public health problem that causes high rates of mortality in the population due to the association with cardiovascular diseases (CVDs) (Go et al., 2004). Thus, CVDs are prevalent in patients with CKD, and subsequent CKD is a significant risk factor for CVDs. Furthermore, patients with CKD are at increased risk for cardiovascular events or death than for progression to end-stage renal disease (ESRD) (Keith et al., 2004). Multiple studies support the notion that patients with renal disease suffer accelerated aging, which precipitates the appearance of pathologies, including CVDs, usually associated with advanced age (Stenvinkel and Larsson, 2013).

Considerable efforts have been made to slow the progression of the disease and improve the quality of life in patients with CKD. New pharmacological strategies do slow the progression of CVDs, and reduce the morbidity and mortality of CKD patients (Merino et al., 2010). Likewise, methods of renal replacement therapy currently offer increased purification capacity and reduced adverse effects. However, the development of CVDs in patients with CKD has not yet been halted. This may be because when CKD is diagnosed, vascular pathology is already advanced and irreversible (Vanholder et al., 2005).

The causes of vascular damage in CKD are exceptionally complex. Among the theories proposed in recent years to explain the high frequency of CVDs in renal patients, one states that senescence of peripheral blood cells (known as immunosenescence) and vascular cells (known as vascular senescence) may be involved in the initiation and perpetuation of vascular pathology that appears early in patients with CKD (Merino et al., 2010, 2011; Carracedo et al., 2013).

Some therapies used successfully in other pathologies, such as CVDs have tested in trials to avoid the progression to ESRD. Statins with a clearly cardioprotective role present non-beneficial results in these CKD patients (Jun et al., 2011). However, recent studies demonstrated that statins in high doses might be improved the renal function (Sanguankeo et al., 2015). In this regard, antiplatelet agents may also be different effects in ESRD (Jun et al., 2011). Moreover, other therapies among them, antioxidants, angiotensin receptor blockers (ARBs) therapies, and angiotensin-converting enzyme (ACE) inhibitors, improve the kidney function in ESRD (Jun et al., 2011).

In this article, we review the cellular and molecular mechanisms, as well as processes involved, in increased risk for CKD-associated CVDs. Summarizing the current state of knowledge on CKD-associated CVDs will aid in early diagnosis and design of novel therapies that will improve the health and quality of life in renal patients.

## Increased Risk for CVDs in Renal Patients, the Current State of the Problem

Cardiovascular diseases are the main cause of morbidity and mortality in CKD patients (Go et al., 2004). This may be because CKD itself is an independent risk factor for CVDs, and is associated with an increased prevalence of traditional and non-traditional risk factors for CVDs. Both estimated glomerular filtration rate (eGFR) and albuminuria are independently associated with cardiovascular outcomes (Matsushita et al., 2015). Even microalbuminuria without a decline in renal function is associated with a twofold to fourfold increase in the risk for CVD (Schiffrin et al., 2007). Furthermore, CVD alone, and numerous risk factors for CVD, exacerbate the progression of this condition and may be risk factors for CKD.

**FIGURE 1:**
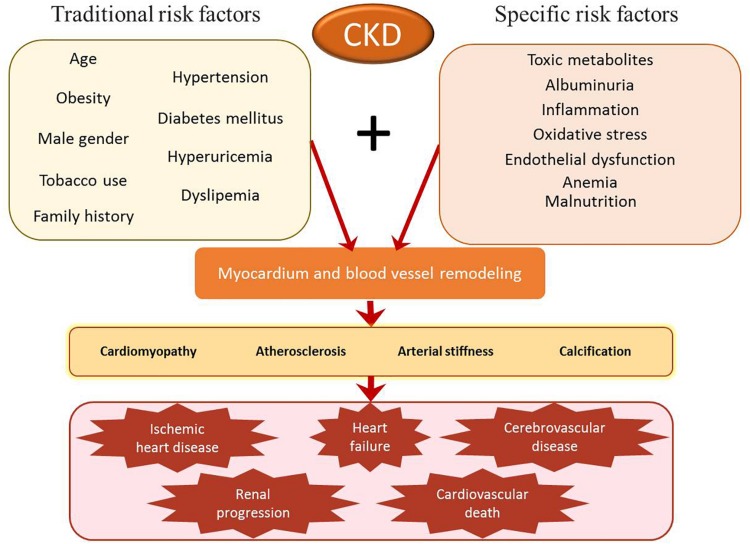
Risk factors for cardiovascular morbidity and mortality in patients with chronic kidney disease (CKD). Traditional risk factors (such as hypertension and diabetes mellitus) and specific CKD risk factors (such as albuminuria or inflammation) lead to remodeling of the myocardium and blood vessels. This process may contribute to the development and progression of cardiomyopathy, atherosclerosis, arterial stiffness, and calcification. Without treatment, these alterations may progress to ischemic heart disease, heart failure, cerebrovascular disease, renal progression, and cardiovascular death.

In patients with CKD, remodeling of the myocardium and blood vessels leads to several cardiovascular complications such as cardiomyopathy, atherosclerosis, arterial stiffness, calcification, and subsequent ischemic heart disease, heart failure, cerebrovascular and cardiovascular death, and progression of renal disease including ESRD (Chen et al., 2018). However, CKD patients present a high incidence of cardiovascular morbidity and mortality that is not fully accounted for by traditional risk factors and those mutual to CVDs and CKD such as age, hypertension, diabetes mellitus, obesity, hyperuricemia, dyslipidemia, tobacco use, family history, and male gender. Specific risk factors for renal impairment include albuminuria, anemia, mineral and bone disorders, malnutrition, toxic metabolites, endothelial dysfunction, inflammation, and oxidative stress (Figure 1).

Patients with CKD have numerous risk factors for the progression of cardiac dysfunction. Several non-traditional risk factors, such as volume overload, anemia, albuminuria, abnormal calcium-phosphate metabolism, inflammation, oxidative stress, and endothelial dysfunction, play critical roles in this process. Left ventricular hypertrophy (LVH) is closely related to the occurrence of heart failure (HF) and is known to be an independent risk factor for mortality (London et al., 2001). Among traditional risk factors, hypertension can be both a cause and a consequence of CKD, while dyslipidemia is another important risk factor for both CVDs and CKD.

## Inflammatory Alterations in Ckd and Their Relationship With the Development of CVDs

Inflammation, which is a new non-traditional risk factor and consequence of reduced kidney function, is highly associated with elevated cardiovascular risk in patients with kidney disease (London et al., 2001). The inflammation transfers to the blood and generates damage in distal tissues in the progression of concomitant diseases such as CVDs (Cottone et al., 2008). Epidemiological and clinical studies have shown a close relationship between markers of inflammation and risk for cardiovascular events (Cottone et al., 2008).

In patients with CKD, traditional cardiovascular risk factors, such as hyperlipidemia and hypertension, are associated with an inflammatory process (Silverstein, 2009). Specific cardiovascular risk factors in CKD patients, such as accumulation of uremic toxins inherent to the use of renal replacement techniques, promote inflammation and are also associated with CVDs (Silverstein, 2009). The chronic, systemic, and low-grade inflammation usually observed in patients with CKD is a long and generalized process that shows no signs of acute inflammation and is mediated by moderate levels of cytokines and inflammatory mediators (Ramirez et al., 2006). The systemic nature and low intensity of this inflammatory response indicate lack of injury and no loss of functionality in these specific tissues; injury and loss of function are distinctive features in both acute and chronic inflammation (Furman et al., 2019). Conversely, the damage generated by systemic inflammation (SI) causes a deterioration in the general state, favoring tumor processes or degenerative diseases such as CVDs (Ungvari et al., 2018).

A similar SI process that is associated with aging is called *inflammaging* (Flynn et al., 2019). SI is considered an adaptive response that causes systemic damage, leading to the development of multiple pathologies, including CVDs (Oishi and Manabe, 2016).

Chronic, low-grade inflammation can be determined by measuring plasma levels of markers such as C reactive protein (CRP) (Sproston and Ashworth, 2018) and cytokines such as IL-6 and TNF-α. In a longitudinal analysis, CRP, measured at baseline during the Modification of Diet in Renal Disease (MDRD) study, was an independent predictor of all-cause and CVD-caused mortality (Levey et al., 1999). In dialysis patients, the association of CRP with mortality was main at low CRP levels, the lower CRP level, the lower mortality risk (Bazeley et al., 2011). Other pro- and anti-inflammatory cytokines, including IL-10 and TNF-α, may also play a role in the development of CKD-associated CVDs (Ekdahl et al., 2017; Mihai et al., 2018).

Although the mechanisms triggering innate immunity and associated inflammation in CKD are still scarce, in spite of some studies have shown that low levels of bacterial endotoxins reached the blood circulatory system in CKD patients, stimulate CKD-associated pathways. Endotoxemia is more prevalent in patients with CKD than in healthy population, and it is linked with CKD-associated CVDs (Ramezani and Raj, 2014; Sirich et al., 2014). Endotoxins, which act as pro-inflammatory stimuli, is linked to endothelial dysfunction, one of the earliest steps in the development of atherosclerosis. Highlighted that there is no data that described the mechanism in which the endotoxins from bacteria get into the circulation of CKD patients. In case the patient undergoes dialysis, the procedure may result the cause of the infection. However, endotoxemia is noted in stages of CKD that precede dialysis (Wu et al., 2011), suggesting that factors unrelated to dialysis contribute to this process.

Disruption of the healthy intestinal barrier, among other factors, may facilitate the passage of bacterial endotoxins into circulation. Two protein-bound uremic toxins, indoxyl sulfate (IS), and p-cresyl sulfate, not removed by conventional dialysis induce inflammation and oxidative stress, causing vascular endothelial cell injury (Ramezani and Raj, 2014) and contributing to progression of renal impairment (Sirich et al., 2014) and CV-related mortality (Wu et al., 2011).

Therefore, physicians need to identify patients at risk and implement early prevention and treatment strategies in these patients. In practice, managing cardiovascular risk in patients with CKD mostly involves reducing modifiable risk factors such as hypertension, dyslipidemia, and disturbance of mineral and bone metabolism. Furthermore, dialysis adequacy and renal transplantation, preferably pre-emptive transplantation, are considered optimal therapies for the reduction of cardiovascular risk in these patients.

## Changes in Cellular Balance of Reactive Oxygen Species Can Cause CVDs in CKD Patients

Oxidative stress appears because of an imbalance between the production and elimination of reactive oxygen species (ROS). Moreover, oxidative stress is considered a hallmark feature of CKD-associated CVDs and contributes to all-cause mortality in this patient population. Also, physiological stress on the body of CKD patients is increased in very early stages of the disease, progresses parallel to the deterioration of renal function and is further exacerbated in patients undergoing dialysis (Liakopoulos et al., 2017, 2019b). In fact, ESRD patients on both hemodialysis (HD) and peritoneal dialysis (PD) manifest significantly enhanced oxidative stress compared with that of predialysis uremic patients (Liakopoulos et al., 2017, 2019a,b). However, the link between the initiation of CKD and oxidative stress remains debated.

Aging *per se* is a physiological process. However, aging is associated with several age-related diseases such as CVDs and CKD. According to the “oxidative stress theory of aging,” aging and age-related diseases are precipitated by chronic oxidative stress (Sies, 1986). Therefore, because incidence of CKD is high in older persons and age is considered the most consistent risk factor for CVDs (O’Hare et al., 2007; Elewa et al., 2012), CKD is often examined in the context of aging (Nitta et al., 2013). Furthermore, other fundamental mechanisms that characterize biological aging (López-Otín et al., 2013), such as cellular senescence, mitochondrial dysfunction, loss of proteostasis, or altered intercellular communication (López-Otín et al., 2013), may also play central roles in the pathogenesis of CKD-associated CVDs (Shimizu and Minamino, 2019). In this context, several studies have shown that CKD promotes cellular senescence and accelerates premature aging via diverse mechanisms in the internal milieu such as redox state perturbations, oxidative damage, inflammation, toxicity, and localized signaling mediated by growth factors (Stenvinkel and Larsson, 2013; Dai et al., 2019).

Low-grade inflammation (involving increased levels of circulating inflammatory mediators in the absence of infection), which is another characteristic feature of aging and age-related diseases (De la Fuente and Miquel, 2009), is a crucial component of CKD (Mihai et al., 2018). Although the precise mechanisms contributing to the high prevalence of inflammation in CKD are not well established, ROS may potentially contribute to inflammation during declining renal function (Cachofeiro et al., 2008). Oxidative stress and inflammation are interlinked processes (De la Fuente and Miquel, 2009). The highly reactive ROS can alter cellular structures and functional pathways. This triggers a vicious cycle in which inflammatory cells, stimulated by cell damage caused by ROS, generate a state of oxidative stress and amplified oxidative damage (Mihai et al., 2018). Therefore, cross-talk between oxidative stress, inflammation, and aging/senescence represents a fundamental triad contributing to the development and progression of CKD-associated CVDs (Cachofeiro et al., 2008; Popolo et al., 2013).

In CKD patients, a pro-oxidative physiologic state leads to oxidative tissue damage (Cachofeiro et al., 2008; Puchades Montesa et al., 2009; Popolo et al., 2013). Indeed, oxidative stress and damage increase in later stages of CKD and become severe in ESRD patients undergoing HD and PD (Liakopoulos et al., 2017,2019a,b). Several oxidative stress parameters gradually increase when renal disease progresses (Table 1), suggesting that a decline in renal function may have a direct effect on the worsening of oxidative stress (Dounousi et al., 2006; Khansari et al., 2009). HD and PD procedures further exacerbate oxidative stress via different underlying mechanisms. In HD patients, several factors, such as duration of dialysis, biocompatibility of dialyzer membrane and dialysate, intravenous iron administration, activation of leukocytes, retention of uremic toxins, or anemia, contribute to the development and accumulation of oxidative products. In addition, the composition of PD dialysate solutions (such as increased osmolarity, low pH, high-glucose content, and presence of lactate buffer) can trigger oxidative stress in PD patients (Liakopoulos et al., 2017,2019a,b; Roumeliotis et al., 2019a). However, PD is considered to be more biocompatible than HD. Indeed, HD shows increased accumulation of pro−oxidants and depletion of antioxidants compared with PD (Liakopoulos et al., 2017,2019a,b; Roumeliotis et al., 2019a).

**TABLE 1:** Factors participating in oxidative stress and damage in patients with CKD, ESRD, and those undergoing HD or DP.

Number of patients	Localization	Parameters	Results	References
31 HC	Whole blood	GSH	HC = mild > moderate > severe CRF = HD	Ceballos-Picot et al., 1996
83 mild CRF	Erythrocytes	GSSG	HC = mild < HD < moderate < severe CRF HC < HD < mild = moderate = severe CRF	
55 moderate CRF	Plasma	GPx	HD < HC < mild < moderate < severe CRF HD < HC = mild = moderate = severe CRF	
47 severe CRF		GSSG	HD < PD < severe < moderate < mild CRF < HC HD > PD > mild = moderate = severe CRF > HC	
18 HD		Cu/Zn-SOD		
30 PD		GSH		
		GSSG		
31 HC	Plasma	AOPPs	HC = mild < moderate < advance CRF	Witko-Sarsat et al., 1998
73 mild CRF		AGE-pentoside	HC < mild < moderate < advance CRF	
53 moderate CRF		MDA	HC < mild = moderate = advance CRF	
36 advanced CRF		GPx	HC > mild > moderate > advance CRF	
61 HC	Serum	TBARS; TAS	HC = CRF	Annuk et al., 2001
37 CRF	RBCs	LOOH	HC < CRF	
		GSH	HC > CRF	
		GSSG; GSSG/GSH	HC < CRF	
70 HC	Plasma	Thiols	HC > CKD	Oberg et al., 2004
60 CKD		Carbonyls	HC < CKD	
		F2-isoprotanes	HC < CKD	
		CRP	HC < CKD	
		IL-6	HC < CKD	
67 HC	Plasma	MDA	HC < CKD	Puchades Montesa et al., 2009
32 CKD (stage 4)		Carbonylated protein	HC < CKD	
		F2-isoprotanes	HC < CKD	
		GSH	HC > CKD	
		GSSG/GSH	HC < CKD	
		CAT; GR; GPx; SOD	HC > CKD	
		8-OH-dG	HC < CKD	
21 HC	PBMNs	ROS	HC < CKD	Fortuño et al., 2005
22 CKD (stages 1 and 2)		NADPH oxidase	HC < CKD	
38 HC	Plasma	Vitamin C	HC > CKD > HD	Gondouin et al., 2015
51 CKD (stages 3, 4 and 5) 50 HD	RBCs	Zn	HC > CKD = HD	
		SOD	HC = CKD > HD	
		XO	HC < CKD < HD	
		MDA	HC = CKD < HD	
1539 HD	Serum	AOPPs	HD > PD	Zhou et al., 2012
556 PD		MDA	HD > PD	

*8-OHdG: 8-hydroxy deoxyguanosine; AGEs: advanced glycation end-products; AOPPs: advanced oxidation protein products; CAT: catalase; CKD: chronic kidney disease; CRP: C-reactive protein; Cu: copper; GPx: glutathione peroxidase; GR: glutathione reductase; GSH: reduced glutathione; GSSG: oxidized glutathione; GSSG/GSH: ratio of oxidized/reduced glutathione; HC: healthy controls; HD: hemodialysis; IL-6: interleukin 6; LOOH: lipid hydroperoxides; MDA: malondialdehyde; PBMNs: peripheral blood mononuclear cells; PD: peritoneal dialysis; RBCs: red blood cells; ROS: reactive oxygen species; Se: selenium; SOD: superoxide dismutase; TAS: total antioxidant status; TBARS: thiobarbituric acid reactive substances; XO: xanthine oxidase; Zn: zinc.*

**FIGURE 2:**
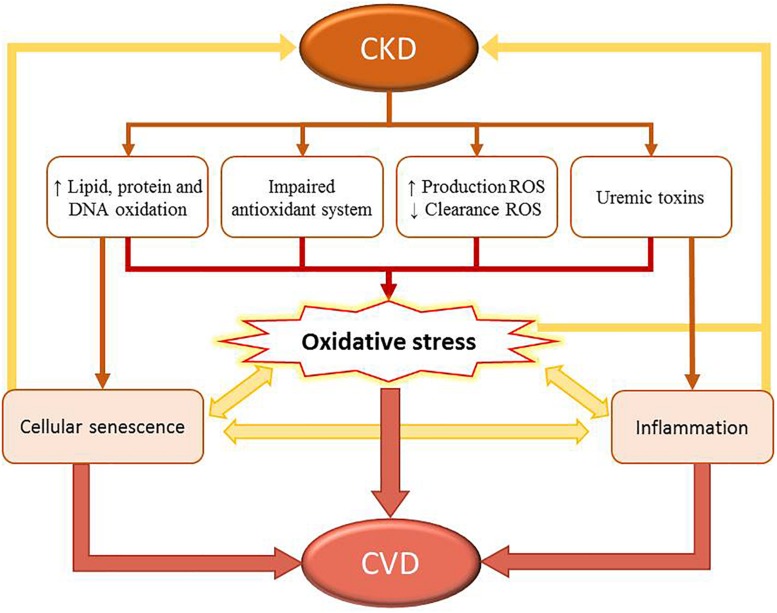
Relationship between oxidative stress and CKD-associated cardiovascular disease (CVDs). CKD leads to increased damage of biomolecules (such as lipids, proteins, and DNA), impairment of the antioxidant system, increased levels of reactive oxygen species (ROS), decreased ROS clearance, and high concentration of uremic toxins in circulation. This process increases the levels of oxidative stress. CKD is also associated with systemic inflammation, mitochondrial dysfunction, loss of proteostasis, altered intercellular communication, and cellular senescence. Combined, these factors contribute to increased levels of oxidative stress in this patient population. However, cellular senescence and inflammation also participate in the development and progression of CVDs.

Table 1 lists clinical studies on triggers of oxidative stress and damage in patients with CKD and ESRD undergoing HD or DP. The underlying mechanisms responsible for oxidative stress in CKD include: increased production and decreased clearance of ROS and other oxidant compounds, such as reactive nitrogen species or chlorinated oxidants; impaired function of antioxidant systems, including both enzymatic [i.e., catalase, superoxide dismutase (SOD), glutathione peroxidase (GPx)], and non-enzymatic [i.e., glutathione (GSH), thioredoxins, and vitamins C, E, and A] systems (Kobayashi et al., 2005; Popolo et al., 2013; Gondouin et al., 2015); increased oxidation of lipids, proteins, and DNA. Indeed, CKD patients show elevated levels of lipid-peroxidation products [such as lipid hydroperoxides and malondialdehyde (MDA), thiobarbituric acid-reactive substances (TBARS), 4-hydroxynonenal (HNE), and F2-isoprostanes], oxidized LDL, protein carbonyls, advanced-oxidation protein products (AOPPs), advanced-glycation end products (AGEs), as well as 8-hydroxy-2′-deoxyguanosine (8-OH-dG) and 8-oxo-7,8-dihydro-2′-deoxyguanosine (8-oxo-dG). These factors contribute to atherosclerotic lesions in these patients (Popolo et al., 2013; Sung et al., 2013; Daenen et al., 2019). Moreover, genetic polymorphisms and mutations in DNA repair genes, such as *FAN1* or *hOGG1*, and DNA repair nucleases, are involved in increased accumulation of damaged nuclear DNA, which contributes to progressive loss of kidney function (Stenvinkel and Larsson, 2013; Sung et al., 2013). Uremic toxins, such as IS, homocysteine, and AGEs, contribute to early senescent of endothelial cells, and also play critical roles in the development of CKD. In CKD patients, progressive deterioration of renal function leads to the accumulation of uremic toxins, which can promote oxidative stress and inflammatory pathways (Sung et al., 2013). For instance, IS induces uncoupling of endothelial nitric oxide synthase (eNOS) and increased expression and activity of nicotinamide adenine dinucleotide phosphate (NADPH) oxidases, leading to increased levels of ROS and endothelial dysfunction in patients with CKD (Yu et al., 2011; Daenen et al., 2019). High IS concentration is a risk factor for CKD-associated CVDs and accelerates the progression of CKD (Yu et al., 2011; Sung et al., 2013). Moreover, IS enhances the production of mitochondrial ROS and downregulates the expression of cellular protective factors by activating NF-κB and related signaling by nuclear factor erythroid-derived 2-like 2 (NRF2), hemeoxygenase-1 (HO-1), and NADPH quinone oxidoreductase-1. In addition to causing increased levels of ROS, this cascade also upregulates the production of pro-inflammatory cytokines (Dou et al., 2007; Zakkar et al., 2009; Masai et al., 2010; Hwang et al., 2019). Moreover, NRF-2 downregulates the expression of proteins involved in mitogen-activated protein kinase (MAPK) pathways, contributing to cellular apoptosis and senescence (Zakkar et al., 2009; Tebay et al., 2015). Figure 2 shows the relationship between oxidative stress and CKD-associated CVDs.

In addition to formation and accumulation of pro-oxidant molecules, CKD patients, especially those undergoing dialysis, can also suffer a considerable downregulation in antioxidative and defensive mechanisms. This is associated with several factors, such as malnutrition, dietary restrictions, or loss of vitamins and trace elements occurring during HD or PD (Liakopoulos et al., 2017, 2019b; Roumeliotis et al., 2019a). Implementing lifestyle interventions, such as administration of exogenous antioxidants, in patients with CKD on dialysis may downregulate the inflammation and oxidative stress associated with CVD-linked morbidity and mortality even in early stages of CKD (Liakopoulos et al., 2019a, b; Roumeliotis et al., 2019b). Indeed, several studies have investigated the possible benefits of exogenous antioxidant administration in both HD and PD patients. Whether vitamins (B, C, D, and E), statins, coenzyme Q-10, N-acetylcysteine (NAC), omega-3 fatty acids, flavonoids, polyphenols, curcumin, green tea, and L-carnitine are beneficial in HD and PD patients remains controversial. Although these data are limited and mainly derived from animal studies or small observational trials, they have generally shown that daily intake of vitamin C and E, NAC, polyphenols, curcumin, or flavonoids, combined with a standard urate-lowering therapy, may act synergistically to ameliorate the deleterious effects of hyperuricemia and oxidative stress in CKD (Liakopoulos et al., 2019a, b; Roumeliotis et al., 2019b). However, further studies in large cohorts of HD and PD patients are necessary to determine the causality between antioxidant supplementation and clinical hard end-points of CVDs and all-cause mortality.

## Immune Alterations in Patients With CKD and Their Relationship With Vascular Pathology

CKD is classically associated with immunodeficiency that contributes considerably to all-cause morbidity and mortality (Cohen and Hörl, 2012). Patients with CKD and those undergoing renal replacement therapy show quantitative alterations in components of the immune system and altered functionality of these components. Numerous studies have focused on relationships between immune-cell senescence and early onset of CKD-associated CVDs.

The number and function of lymphoid cells suffer a decrease associated with the loss of the renal function. This is characterized by loss of thymic function, shortening of telomeres, and expansion of memory T-cell populations, which is compatible with the concept of premature immune aging (Georgin-Lavialle et al., 2010).

**FIGURE 3:**
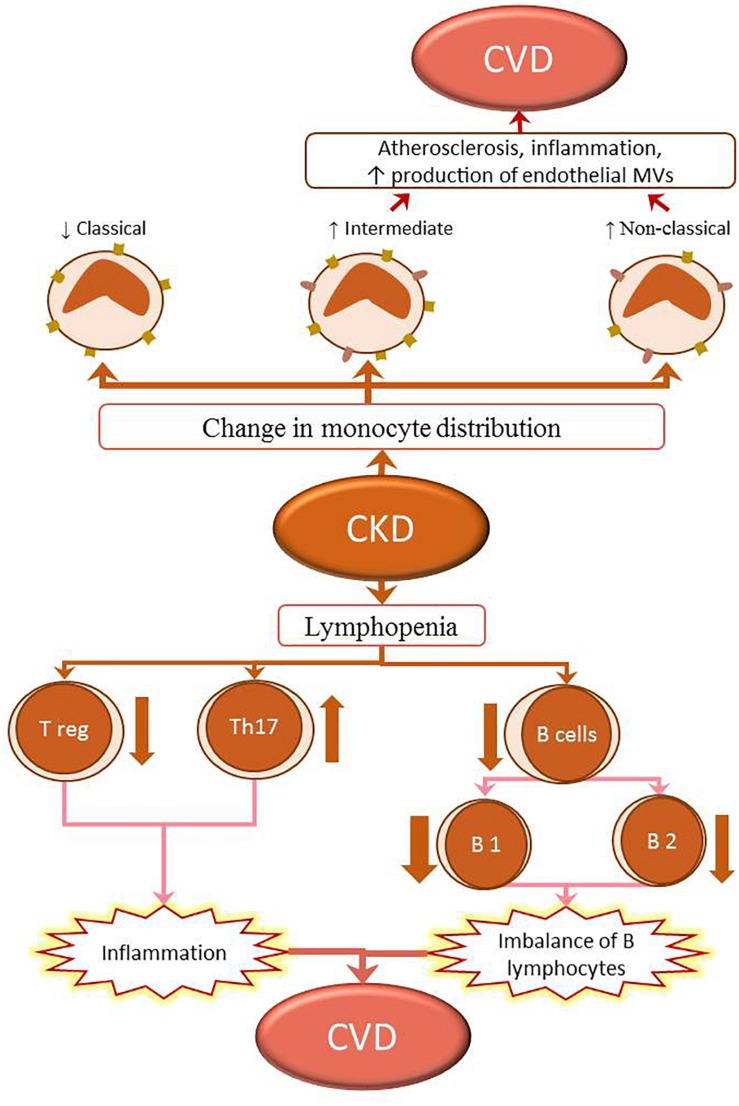
Changes in the immune system in CKD lead to the development of CVD. The populations of classical (CD14++CD16-) monocytes in CKD decrease, while those of intermediate (CD14++CD16+) and non-classical (CD14+CD16+) monocytes increase. The intermediate (CD14++CD16+) and non-classical (CD14+CD16+) monocytes promote inflammation and proatherogenic milieu by upregulating the expression of adhesion molecules and production of microvesicles (MVs) in the endothelium; this contributes to the development of CVD in patients with CKD. Lymphopenia of both T and B lymphocytes is observed in CKD. The T lymphopenia is caused by the reduction of T regulatory cells (Treg), contrariwise the increase of pro-inflammatory T helper 17 lymphocytes (Th17). These changes in proportion of regulatory cells to proinflammatory cells can lead to inflammation. B lymphopenia is caused by decreased numbers of B1 and B2 cells. The numbers of B1 lymphocytes generally show a greater decline than those of B2, resulting in a B-lymphocyte imbalance. These two processes also participate in the development of CKD-associated CVD.

Total lymphopenia is a marker of overall mortality in patients on hemodialysis (Pahl et al., 2010; Saad et al., 2014). Patients with ESRD or those undergoing renal replacement therapy show a decreased response to specific pathogens and vaccines. These patients also show an increased susceptibility to intracellular pathogens and increased incidence of tumors compared with the general population (Eleftheriadis et al., 2007). Different subtypes of lymphocyte populations and immunoglobulins play ambivalent roles in atherosclerosis. Some lymphocytes and immunoglobulins (T-helper lymphocytes, natural killer cells, T-cytotoxic lymphocytes, and B2 cells, and IgG- and IgE-secreting cells) play a pro-atherogenic role, while others [Tregs, B1a cells, innate response activator (IRA) B cells, Bregs, and natural IgM-secreting cells] show protective functions in healthy subjects (Tsiantoulas et al., 2015). A previous study has examined the roles of different subtypes of T lymphocytes and their cytokines in patients with CVDs and ESRD or those undergoing renal replacement therapy (Danyan et al., 2013). In patients with CKD, studies have focused on the roles of T lymphocytes, increased numbers of Th17 cells, and their proatherogenic activity (Danyan et al., 2013) (Figure 3).

The roles of B cells in the development of CKD-associated CVDs are unclear. Patients undergoing hemodialysis frequently develop lymphopenia B. This is due to increased cellular apoptosis that results from decreased Bcl-2 expression, and resistance to interleukin-7 (IL-7) and B-cell-activating factor of the TNF family (BAFF), which is necessary for the differentiation and survival of B lymphocytes (Fernández-Fresnedo et al., 2000; Pahl et al., 2010). B cells are classified into subtypes B1 (B1a and B1b) and B2 (Figure 3). Patients on hemodialysis have decreased numbers of both B1 (atherogenic B1a) and B2 (proatherogenic) lymphocytes, although the reduction in B1 lymphocytes is generally more significant (Pahl et al., 2010). This milieu causes a chronic imbalance favoring pro-endogenous B lymphocytes, which increase the incidence of cardiovascular pathology.

A recent study, examining the roles of different lymphocyte populations in the mortality of hemodialysis patients in Spain, has shown that the number of B lymphocytes is an independent risk factor for overall mortality in this patient population. The study has shown that cardiovascular pathology is the leading cause of mortality in these patients, and that a relationship exists between decreased B-cell numbers and mortality from cardiovascular causes. The leading cause of mortality in this study was cardiovascular, and in this aspect, there was a relationship between the decrease in B cells and the mortality of cardiovascular causes, confirming this data with the determination of B lymphocytes at 12 months of follow-up. Conversely, patients with a CD56+ lymphopenia show a higher prevalence of mortality from infectious causes (Molina et al., 2018).

Patients with ESRD currently show high mortality; therefore, multiple parameters have been evaluated to predict the mortality of this population (Floege et al., 2015). CD19 lymphopenia may be a new marker for predicting the risk for morbidity and mortality in patients on hemodialysis. Therefore, accurate and timely diagnosis of CD19 lymphopenia in patients with ESRD and those undergoing dialysis may lead to new therapeutic targets against CKD.

The three subpopulations of peripheral blood monocytes, identified in the innate immune system, are: classical monocytes (CD14++/CD16-), existing mainly in healthy subjects; intermediate monocytes (CD14++/CD16+); and non-classical monocytes (CD14+/CD16+). Intermediate monocytes (CD14++/CD16+) may be involved in the development of atherosclerosis both in the general population and in patients with CKD (Georgin-Lavialle et al., 2010; Merino et al., 2011). CD14+/CD16+ monocytes are inflammatory cells with high capacity to produce pro-inflammatory cytokines and possess a robust dendritic-cell – like function. In peripheral blood of elderly individuals and patients with CKD, non-classical monocytes accumulate because of their increased resistance to apoptosis (Ramírez et al., 2005). CD14+/CD16+ cells express elevated levels of adhesion molecules and chemokines and show high adhesion capacity on endothelial cells (ECs). In ECs, CD14+/CD16+ cells induce increased expression of angiogenic factors and production of microvesicles (MVs), which are instrumental in induction of vascular damage (Merino et al., 2011). Therefore, accumulation of CD14+/CD16+ monocytes in peripheral blood plays a prominent role in inducing and perpetuating the inflammatory process in elderly individuals and patients with CKD and may have a direct relationship with the development of CVDs (Figure 3).

## Cellular Alterations in the Vascular System of Patients With CKD

The sustained microinflammatory milieu that persists in patients with CKD may be related to increased endothelial damage (Merino et al., 2010; Cohen and Hörl, 2012). Changes in endothelium are considered main factors indicating vascular deterioration in the early detection of CVDs (Heine et al., 2012). Endothelial aging, associated with age or induced by CKD, favors endothelial dysfunction (Ramírez et al., 2005). *In vitro* studies have shown that uremia determines a state of senescence in ECs, which is also associated with chronic inflammation (Carracedo et al., 2013). Therefore, both endothelial damage/dysfunction and inflammation likely play important roles in the initiation and progression of cardiovascular complications associated with CKD (Ross, 1999).

Endothelial damage is associated with increased production of extracellular vesicles (EVs). EVs serve as a signaling system between the factors involved in the function and homeostasis of the organism (Ekdahl et al., 2017). EVs are classified into three types based on their biogenesis: exosomes, MVs, and apoptotic bodies (Yáñez-Mó et al., 2015; van Niel et al., 2018). EVs are produced by most cell types, especially when cells are in contact with liquids. EVs are also produced under physiological and pathological conditions, although their number and molecular content differ under both conditions; indeed, of the potential use of EVs as pathological markers is based on these differences (Yuana et al., 2013). Aging and stress also induce changes in EVs. EVs are involved in the origin and development of various pathologies, and may be used as potential biomarkers or therapeutic agents in patients with CVDs (Amosse et al., 2017; Jansen et al., 2017; Dickhout and Koenen, 2018). EVs are involved in the development of renal dysfunction (Karpman et al., 2017; Lv et al., 2019), and can, therefore, be used in the diagnosis of, and therapy against, these diseases (Zhang et al., 2016). Elimination of EVs is currently used as a therapeutic strategy (Tang and Liu, 2019). Endothelial MVs can serve as useful biomarkers of CKD (Carmona et al., 2017a) because endothelial damage is associated with CKD, and the release of EVs is enhanced during endothelial injury. Uremic toxins (such as IS) also induce a release of endothelial MVs (Carmona et al., 2017b). Not only are plasma EVs modified in kidney diseases, but the release of EVs from renal cells (epithelial cells, podocytes, and tubular cells) is also altered. These kidney-derived EVs can be isolated from urine and used as biomarkers of CKD (Lv et al., 2019). Proteomic analyses have shown differences in urinary EVs obtained from patients with kidney diseases (Bruschi et al., 2019). These differences can be used to establish stages in the development of kidney (Stokman et al., 2019). In addition to serving as biomarkers, blood and urinary MVs play functional and therapeutic roles in renal diseases (Erdbrügger and Le, 2016; Kwon, 2019). For example, blood EVs influence renal physiology via intranephron communication (Lv et al., 2019), and their antibacterial (Hiemstra et al., 2014) and protective effects (Bruno et al., 2009, 2016; Rovira et al., 2017) have been demonstrated in kidney diseases. ROS are important in the origination and development of CKD, while MVs play antioxidative roles in this condition (Bodega et al., 2019).; However, the specific roles of blood and urinary MVs in redox dysregulation occurring in CKD have not been determined.

## MVs microRNAs Mediate CKD-Associated CVDs

MicroRNAs (miRNAs) are small, approximately 22-nucleotide long strands of small non-coding RNAs that regulate post-transcriptional processes (Ramshani et al., 2019; Rios et al., 2019). Unlike RNA, miRNAs show high stability in fluids and can be reliably detected in the blood (Mitchell et al., 2008). MiRNAs levels differ in healthy and diseased tissues (Liu et al., 2019) and play a critical role in the maintenance of cardiovascular homeostasis (Pfeifer et al., 2015). EV-associated miRNAs have been studied extensively (Liu et al., 2019; Ramshani et al., 2019). Numerous studies have shown that miRNAs can be used as valuable pathological and therapeutic biomarkers in both cells and EVs (Bekris and Leverenz, 2015; Lei et al., 2017). Therefore, miRNAs may act as master regulators of signaling pathways involved in CKD-associated CVDs. Moreover, different profiles of EVs miRNAs in injured cells can be used as diagnostic markers in various pathologies such as cancer (Chen et al., 2019), viral infections (Yoshikawa et al., 2019), lung diseases (Chen et al., 2017), ischemic stroke (van Kralingen et al., 2019), CVDs (Bellin et al., 2019), and especially CKD-associated CVDs (Jansen et al., 2017; Blaser and Aikawa, 2018). Cells from vascular walls may selectively package miRNAs as molecular cargo in EVs (Rios et al., 2019).

CKD-associated CVDs are associated with premature aging. Accelerated aging of the vascular wall induces a senescent phenotype in vessel cells, and particularly in ECs (Carracedo et al., 2018a, b). EVs, released by senescent cells, are involved in mechanisms driving the acquisition of this senescent phenotype in ECs, which promotes the progression of CVDs. miRNA-loaded EVs induce numerous injury-associated responses in other vascular cells, leading to the development of CVDs (Carracedo et al., 2018a). MiRNAs are essential to the homeostasis and maintenance of EC activity (Ballantyne et al., 2019). Some of the miRNAs involved in endothelial and vascular smooth muscle cell (VSMCs) homeostasis are described in Table 2.

**TABLE 2:** Highlighted miRNAs mediate homeostasis of the vascular wall.

MiRNAs	Expression	Target	Function	Inverse (negative) regulated by	References
**Healthy ECs**					
miR-155	↓(Down)	Endothelial nitric oxide synthase (eNOS)	Endothelium, vascular relaxation	Inflammatory factor (TNF-α) (↑ miR-155)	Ballantyne et al., 2019; Chen et al., 2019
miR-126	↑(Up)	Vascular endothelial growth factor A (VEGF-A)	Angiogenesis	Sprouty-related EVH1 domain 1 (SPRED-1) (↓ miR-126)	Alique et al., 2019; Ballantyne et al., 2019
**Physiological VSMC phenotype**					
miR-21	↑(Up)	Phosphatase and tensin homolog (PTEN) B-cell lymphoma 2 (Bcl-2) expression	SMC proliferation and survival (anti-apoptotic)	Programmed cell death protein 4 (PDCD4)	Maegdefessel et al., 2015; Ballantyne et al., 2019
miR-143/145 cluster	↑(Up)	Transcription factors such as Kruppel-like factor (KLF)-4, (miR-145), KLF5, and ELK-1	VSMCs contractility and proliferation	Angiotensin-converting enzyme (ACE), KLF-4, myocardin	Maegdefessel et al., 2013; Maegdefessel et al., 2015; Metzinger-Le Meuth et al., 2017; Ballantyne et al., 2019; Vacante et al., 2019

MiRNAs modulation is critical in numerous disorders. For instance, miR-155 and miR-223 are responsible for the imbalance between calcium and phosphate that is observed in the vessels of patients with CKD. Moreover, patients with CKD show a dysregulation of miR-155 and miR-223, which causes their bones to lose calcium and phosphate; these ions are then accumulated in the vascular wall (Metzinger-Le Meuth et al., 2017). Recently, miR-223 has implied in the gene program of osteoclastogenesis and macrophage differentiation during the vascular calcification. This is a common complication in CKD that affects the vascular system due to pathological deposition of calcium and phosphate in the vessels (Metzinger-Le Meuth et al., 2017). Also, Ulbing et al. (2017) have shown that the expression of seric miR-223 is decreased in a murine model of CKD; decreased levels of seric miR-223 are also used to confirm CKD stage 4 and 5 diagnosis in patients with CKD. In this context, the levels of miR-155, miR-125b, and miR-145 are also decreased in stage 3-5D CKD patients (Chen et al., 2013).

Microvesicles (Hunter et al., 2008) and exosomes (Valadi et al., 2007) may actively play pathological roles in the development and progression of CVDs because they act as carriers of various miRNAs. miRNAs can be carried by RNA-binding proteins such as Argonaute 2 (Arroyo et al., 2011), or lipoprotein complexes such as HDL (Vickers et al., 2011). MiR-223 could be delivery, in part linked to HDL, toward to intima and media wall and could be transferred to ECs suppressing the expression of intercellular adhesion molecule 1 (ICAM-1) (Metzinger-Le Meuth et al., 2017). Accordingly, the results of our previous study show that both strands of miR-126 are encapsulated in endothelial MVs, and that vascular endothelial repair is mediated by miR-126 and HIF-1α (Alique et al., 2019). Results reported that miRNAs are involved in the senescence phenotype acquisition (Alique et al., 2017, 2019; Carracedo et al., 2018b). Several miRNAs–mediated pathological effects are described in Table 3.

**TABLE 3:** MiRNAs involved in pathologies of the vasculature.

MiRNAs	Expression	Pathology	Effect	Via	References
**Damage ECs**					
miR-125b	↑(Up)	Stimulation or ischemia	Inhibition of *in vitro* tube formation	↓ VE-cadherin VEGF	Vosgha et al., 2018; Ballantyne et al., 2019
miR-126	↓ (Down)	Aging	Senescence	Hypoxia-inducible factor 1 (HIF-1)-α	Alique et al., 2019
		CKD	Endothelial dysfunction	CXCL12 V-CAM 1	Metzinger-Le Meuth et al., 2017
miR-34b-5p	↓ (Down)	Cancer	Proliferation and angiogenesis	VEGF-A	Maroof et al., 2017
miR-205	↑(Up)	Epithelial-to-mesenchymal transition (EMT) and cancer	Inhibition of tumor growth	VEGF-A	Vosgha et al., 2018
**VSMCs phenotype regulation**					
miR-21	↓ (Down)	Abdominal aortic aneurysms and atherosclerosis (unstable plaques in humans)	Antiproliferative	RE1-silencing transcription factor (REST)	Barwari et al., 2018
miR-143/145 cluster	↓ (Down)	Vascular injury (vascular remodeling), hypertension, atherosclerosis, and pulmonary-arterial hypertension	VSMC differentiation and phenotypic switch	↓ Jag-1/Notch ↓ SRF/Myocardin	Vacante et al., 2019
**Alteractions in ECs and VSMCs**					
miR-155	↓ (Down)	CKD osteoclastogenesis (vascular calcification)	Differentiation of monocytes/macrophages into osteoclast-like cells	↓TNF-α	Metzinger-Le Meuth et al., 2017
miR-223	↑(Up)	CKD (vascular calcification, osteoclastogenesis)	Transdifferentiation of VSMCs into an “osteoblast-like” phenotype	RhoB/MEF2C/SMαA	Metzinger-Le Meuth et al., 2017

MiR-145 is upregulated in the plasma of patients with stable coronary artery disease, stable or unstable angina, and acute myocardial infarction (Vacante et al., 2019). Nonetheless, EVs contain-associated miR-143 acts as a mediator of communication between ECs and VSMCs in CVDs. miR-143 in particular is associated with vascular remodeling in pulmonary arterial hypertension (Vacante et al., 2019), while the miR-143/145 cluster, released from VSMCs, modulates angiogenesis and EC proliferation (Climent et al., 2015; Vacante et al., 2019). These findings indicate that miR-143 and miR-145 regulate VSMCs phenotype and mediate the development of vascular diseases. Therefore, miRNA expression may be used as a biomarker of CKD-associated CVDs, while modulation of this expression can be used to develop novel therapeutic approaches against these diseases.

MiR-21 is linked with VSMCs physiological proliferation (Ballantyne et al., 2019) and kidney fibrosis because the abrogation of miR-21 has been described as a protector against the development of fibrosis (Metzinger-Le Meuth et al., 2017). Furthermore, miR-21 is upregulated in ECs collected from atheroma plaques and in progenitor cells obtained from patients with coronary artery disease (Pfeifer et al., 2015). Hence, the elimination of miR-21 could be a useful therapy to avoid the development of kidney fibrosis, whereas this effect could be hazardous for the normal VSMCs proliferation in the vessels. In this regard, the developing localized therapies in the kidney may reduce the risk of adverse events in this treatment approach (Barwari et al., 2018).

## Conclusion and Future Perspectives

In this review, we discussed the cellular and molecular factors involved in accelerated senescence that is observed in patients with CKD. The aging process that occurs due to uremia is associated with numerous changes at the cellular and molecular level, which coincide with changes observed during the physiological aging process. These changes may explain some of the complications that typically occur in patients with CKD and CKD-associated CVDs. Expanding our understanding of the factors and molecules involved in accelerated senescence will serve to identify possible targets associated with this process. This will lead to improved methods of diagnosis and monitoring of these patients. Understanding the similarities between accelerated senescence and normal physiological aging will help establish new treatments. Further studies are needed to assess whether treatments aimed at delaying physiological aging can be applied in CKD.

## Author Contributions

JC chose the topic for this review and enlisted MA, CV, GB, NC, EM, MP, PS, and RR as coauthors. JC, MA, and RR defined the manuscript topic and outline conceived. All authors contributed to the writing and editing of this manuscript, and approved the final version of the manuscript.

## Conflict of Interest

The authors declare that the research was conducted in the absence of any commercial or financial relationships that could be construed as a potential conflict of interest.
